# Nutritional Composition, Glycemic Index, Antioxidant and Anti-Inflammatory Activities of Five Monofloral Honeys from Qassim, Saudi Arabia: A Component–Effect Relationship Study

**DOI:** 10.3390/foods15132263

**Published:** 2026-06-24

**Authors:** Sarah A. M. Alnafisah, Sami A. Althwab, Rehab F. M. Ali

**Affiliations:** Department of Food Science and Human Nutrition, College of Agriculture and Food, Qassim University, Buraydah 52571, Saudi Arabia; 451213978@qu.edu.sa (S.A.M.A.); thaoab@qu.edu.sa (S.A.A.)

**Keywords:** monofloral honey, Qassim region, antioxidant activity, anti-inflammatory activity, glycemic index, phenolic compounds, component-effect relationship, antioxidants

## Abstract

**Background:** Honey possesses anti-inflammatory, antimicrobial, and wound-healing properties due to its complex mixture of carbohydrates, phenolics, flavonoids, vitamins, and minerals. However, comprehensive data on monofloral honeys from Saudi Arabia’s Qassim region are lacking. **Objective:** This study evaluated the nutritional composition, glycemic index, antioxidant and anti-inflammatory activities of five Qassim honeys (Talh, Keina, Samr, Berseem, Ashr) and identified chemical components responsible for their therapeutic potential. **Methods:** Physicochemical parameters, sugar profiles, minerals, vitamins, total phenolic content (TPC), total flavonoid content (TFC), diastase activity, and HMF were analyzed. Antioxidant activity was assessed by DPPH and ABTS assays, anti-inflammatory activity by BSA denaturation inhibition, and in vitro glycemic index by simulated digestion. Statistical comparisons used one-way ANOVA with Fisher’s LSD (*n* = 5). **Results:** All honeys met international quality standards (moisture < 20%, HMF < 40 mg/kg, F + G > 60 g/100 g). Samr honey showed the highest TPC (890 mg GAE/kg) and TFC (226 mg QE/kg). Ashr and Berseem exhibited the highest DPPH scavenging (92% and 91%). Samr was the most potent ABTS scavenger (IC_50_ 26.7 μg/mL). Ashr displayed the strongest anti-inflammatory activity (86.9%), followed by Berseem (72.0%). All honeys had low glycemic index (51–55). Talh and Samr (Acacia-derived) were richest in K, Mg, P, Fe, and Zn; Keina (Eucalyptus) was highest in Ca; Berseem (clover) had the lowest mineral content. Samr honey contained the highest levels of vitamin C and B vitamins. **Conclusions:** The five Qassim monofloral honeys possess distinct nutritional and bioactive profiles. Samr honey is exceptionally rich in phenolics, flavonoids, and B vitamins, contributing to high antioxidant capacity. Ashr and Berseem honeys showed remarkable anti-inflammatory activity. All honeys are low-glycemic and meet quality standards. These findings support their use as functional foods and provide a basis for botanical authentication and quality control.

## 1. Introduction

Honey has been documented as the oldest traditional medicine, effective in suppressing inflammation, enhancing wound repair, and promoting rapid autolytic debridement [1]. Its nutritional and therapeutic properties are determined by a complex mixture of carbohydrates (75–80%, mainly fructose and glucose), proteins, enzymes, vitamins, minerals, and bioactive phytochemicals, primarily phenolic compounds and flavonoids [2]. The botanical and geographical origin profoundly influences honey’s composition and therapeutic potential [3,4]. For example, Talh honey (Acacia nilotica) is rich in quinic acid, gallic acid, and flavonoids; Keina (Eucalyptus) honey contains epigallocatechin gallate and taxifolin; and Manuka honey possesses methylglyoxal as a unique non-peroxide antibacterial agent [3,5,6]. These honeys exhibit potent antioxidant, antimicrobial, anti-inflammatory, and wound healing activities. Talh honey has demonstrated anti-obesity and hepatoprotective effects in animal models [7] and has been reported to accelerate diabetic foot ulcer healing clinically [1]. Keina (Jarrah) honey shows broad-spectrum antibacterial activity through hydrogen peroxide production [6,8]. Nevertheless, these effects may vary depending on honey composition, processing methods, and storage conditions. Therefore, monofloral honeys represent valuable natural products, provided that rigorous quality control is maintained [2,4].

The Qassim region (central Saudi Arabia) is characterized by an arid climate with distinct seasonal flowering, supporting diverse nectar-producing plants such as Acacia, Eucalyptus, Trifolium, and Calotropis [2,5]. Honey from Qassim is highly valued in local traditional medicine for wound healing, respiratory ailments, and gastrointestinal disorders [1,5,7]. However, rigorous scientific characterization of Qassim’s monofloral honeys remains limited [2,8], and no study has compared their nutritional and functional properties side-by-side [8]. Therefore, investigating Qassim honeys is not only regionally important for authentication and quality control but also provides a model for understanding how botanical origin in semi-arid ecosystems influences honey bioactivity [2,4,9].

In this context, five distinct monofloral honeys produced in the Qassim region of Saudi Arabia namely Talh, Keina, Samr, Berseem, and Ashr–were selected for comprehensive analysis. The rationale for selecting these five specific monofloral honeys is based on (i) their traditional medicinal use in the Qassim region, (ii) their commercial availability, and (iii) the lack of prior comparative studies, particularly for Ashr (*Calotropis procera*) honey. Talh honey (Acacia nilotica) is a unifloral honey produced by Apis mellifera jemenitica in southern Saudi Arabia. Its dark amber color reflects high phenolic and flavonoid content, including gallic, vanillic, and quinic acids [3]. It has been reported to exhibit strong antibacterial activity against multidrug-resistant Pseudomonas aeruginosa and MRSA [9,10], anti-obesity and hepatoprotective effects [7], and accelerated wound healing in diabetic foot ulcers [1]. Keina (Eucalyptus) honey is aromatic with potent antimicrobial, antioxidant, and respiratory soothing properties, attributed to phenolic compounds and hydrogen peroxide [6,11,12]. Traditionally, it soothes asthma and throat infections [12,13]. Samr honey (Acacia tortilis/Vachellia tortilis) has low moisture (≈14.7%), low HMF, and high total phenolics (1624–2898 mg GAE/kg) with flavonoids such as quercetin, rutin, and catechin [14,15]. It is rich in vitamin C [16] and exhibits potent antimicrobial and anti-inflammatory effects [14,17,18,19]. Berseem honey (Trifolium alexandrinum) is widely cultivated as a forage crop. It contains significant minerals and bioactive compounds such as quercetin, rutin, and catechin [14,20,21]. Berseem honey shows potent antimicrobial activity against MRSA, E. coli, P. aeruginosa, and Salmonella, with non-peroxide components contributing ≈88% of activity [9,22,23], as well as anti-inflammatory effects via COX-1/COX-2 inhibition and NF-κB blockade [24,25]. Ashr honey (Calotropis procera) is produced from a drought-resistant shrub used in traditional Bedouin medicine. Recently, Raweh et al. [4] provided the first peer-reviewed physicochemical characterization, confirming it meets international standards. However, no studies have yet investigated its bioactive composition or therapeutic activities.

Despite the recognized medicinal value of these honeys, no comprehensive study has compared their nutritional composition, glycemic index, and multi-biological activities (antioxidant, anti-inflammatory) in relation to their chemical profiles. Moreover, the Qassim region of Saudi Arabia produces unique honeys that have not been systematically characterized. Therefore, the present study aimed to evaluate the nutritional value, glycemic index, and multi-biological activities of five monofloral honeys from Qassim, and to identify the chemical components responsible for their therapeutic potential through correlation analysis of their composition (sugars, minerals, vitamins, phenolics, flavonoids) and biological effectiveness. The novelty of this work lies in (i) the first comparative analysis of these five specific Qassim honeys including Calotropis procera (Ashr) honey, (ii) the integration of multiple biological endpoints (antioxidant, anti-inflammatory, glycemic) with detailed chemical profiling, and (iii) the use of multivariate statistical methods (PCA, hierarchical clustering) to establish component–effect relationships. In this study, component–effect relationship refers to the quantitative associations between individual or groups of chemical constituents (sugars, phenolics, flavonoids, minerals, vitamins) and the corresponding biological activities (antioxidant, anti-inflammatory, glycemic index). These relationships are explored using multivariate statistical tools, including principal component analysis (PCA) and hierarchical clustering heatmaps, which reveal which components drive which biological effects across the five honey types.

## 2. Materials and Methods

### 2.1. Materials

Five local honey samples were collected directly from beekeeping apiaries in the Qassim region of Saudi Arabia. The samples were identified based on their botanical origin as follows: Talh honey from *Acacia gerrardii* (production date: 27 June 2025); Keina honey from *Eucalyptus* spp. (production date: 27 May 2025); Samr honey from *Vachellia tortilis* (syn. *Acacia tortilis*) (production date: 8 June 2025); Berseem honey from *Trifolium alexandrinum* (production date: 15 July 2025); and Ashr honey from *Calotropis procera* (production date: 20 August 2025). All samples were collected during the 2025 flowering season, stored in sterile glass containers, and kept at 4 °C in the dark until analysis to preserve their physicochemical and bioactive properties. Botanical origin was confirmed by melissopalynological analysis according to the method of Louveaux et al. [12]. Briefly, 10 g of honey was dissolved in 20 mL of distilled water at 40 °C and centrifuged at 3000× *g* for 15 min. The sediment was mounted on microscope slides using glycerin-gelatin. At least 500 pollen grains were counted per sample. Pollen frequency was classified as dominant (>45%), secondary (16–45%), important minor (3–15%), or minor (<3%). All five samples showed dominant pollen from their respective floral sources: Talh (*Acacia gerrardii*, 52%), Keina (*Eucalyptus* spp., 58%), Samr (*Vachellia tortilis*, 55%), Berseem (*Trifolium alexandrinum*, 61%), and Ashr (*Calotropis procera*, 48%).

### 2.2. Methods

All chemicals and reagents used in this study, including Folin‑Ciocalteu reagent, sodium carbonate, aluminium chloride, sodium nitrite, DPPH, ABTS, potassium persulfate, bovine serum albumin (BSA), pepsin, Carrez solutions I and II, sodium hydroxide (NaOH), nitric acid (HNO_3_), hydrogen peroxide (H_2_O_2_), acetonitrile, methanol, trifluoroacetic acid (TFA), sucrose, glucose, fructose, gallic acid, quercetin, and certified reference solutions for mineral elements, were obtained from Sigma‑Aldrich (St. Louis, MO, USA) unless otherwise specified.

#### Physicochemical Analyses of Honey

Color Measurement

Color parameters of the honey samples were determined using a Minolta CR-400 colorimeter (HunterLab, Reston, VA, USA). Prior to measurement, the instrument was calibrated against a standard white plate. The color indices recorded were L* (lightness: 0 = black, 100 = white), a* (green–red scale: –60 to +60), and b* (blue–yellow scale: –60 to +60). Chroma (C) and hue angle (H°) were calculated from the L, a, and b* values according to Saxena et al. [26].

Moisture Content

Moisture content was determined using an Abbe refractometer (ATAGO Co., Ltd., Tokyo, Japan) according to AOAC method 969.38 [27]. The refractive index of each honey sample was measured at room temperature, and the corresponding moisture percentage was calculated using a standard conversion table.

Total Carbohydrates

Total carbohydrate content (g/100 g) was calculated by difference according to the following formula [27]:

Total carbohydrates = 100 − (moisture + ash).

where moisture, and ash contents are expressed as percentages (g/100 g).

Ash Content

Ash content (g/100 g) was determined by incinerating approximately 5 g of honey in a pre-weighed porcelain crucible. The sample was first heated gently on a hot plate until complete carbonization, then transferred to a muffle furnace and incinerated at 550 °C until a constant white ash was obtained. The crucible was cooled in a desiccator and reweighed. Ash content was calculated as the percentage of the residual weight relative to the initial sample weight [27].

pH and Total Acidity

pH and total acidity were determined according to AOAC method 962.19 [27]. For pH measurement, a 10% (*w*/*v*) honey solution was prepared using deionized water, and the pH was measured using a calibrated pH meter (Thermo Scientific™ Orion Star™ A111 Benchtop pH Meter, Thermo Fisher Scientific, Waltham, MA, USA). Total acidity (expressed as meq kg^−1^) was determined by titrimetric method: 10 g of honey was dissolved in 75 mL of CO_2_-free distilled water, and the solution was titrated with 0.05 N sodium hydroxide (NaOH) to an endpoint of pH 8.5. The volume of NaOH consumed was used to calculate the total acidity.

Sucrose, Glucose, and Fructose Contents

Sucrose, glucose, and fructose contents (g/100 g) were determined by high-performance liquid chromatography (HPLC) according to AOAC method 977.20 [27]. Honey samples were dissolved in deionized water, filtered through a 0.45 μm membrane filter, and injected into an HPLC system (Agilent Technologies, Santa Clara, CA, USA) equipped with a refractive index detector (RID). Separation was achieved using an amino (NH_2_) column with an isocratic mobile phase of acetonitrile:water (75:25, *v*/*v*) at a flow rate of 1.0 mL/min. Sugar concentrations were quantified by comparison with external standards of sucrose, glucose, and fructose.

Mineral Element Composition

Mineral elements (Cu, Fe, Mn, Zn, Ca, Na, K, Mg, P) were determined using inductively coupled plasma-optical emission spectrometry (ICP-OES). Approximately 2 g of each honey sample was digested in a mixture of concentrated HNO_3_ and H_2_O_2_ (5:1, *v*/*v*) on a hot plate until a clear solution was obtained. The digest was diluted to 25 mL with deionized water and filtered. Analysis was performed using an Agilent 5100 Synchronous Vertical Dual View (SVDV) ICP-OES equipped with an Agilent Vapor Generation Accessory VGA 77 (Agilent Technologies, Santa Clara, CA, USA). Concentrations of the elements were quantified against standard calibration curves prepared from certified reference solutions (Sigma‑Aldrich, St. Louis, MO, USA). Results were expressed in mg/kg (wet weight basis).

Vitamin C, B1, B2, B3, and B6 Contents

Water-soluble vitamins (C, B1, B2, B3, B6) were quantified by high-performance liquid chromatography (HPLC) using an Agilent 1260 series system (Agilent Technologies, Santa Clara, CA, USA). Separation was achieved on a Synergi™ Hydro-RP 80 Å column (4.6 × 250 mm, 4 μm particle size) (Phenomenex, Torrance, CA, USA). The mobile phase consisted of water containing 0.01% trifluoroacetic acid (TFA, pH 2.9) as solvent A and methanol as solvent B, at a flow rate of 1.5 mL/min. The gradient program was as follows: 90% A at 0 min, 80% A at 1 min, 60% A at 4 min, and returning to 90% A from 8 to 10 min. Detection was performed at 270 nm with an injection volume of 5 μL. For sample preparation, 2 g of honey were dissolved in 10 mL of 70% methanol, vortexed for 5 min, sonicated for 5 min, centrifuged at 3000× *g* for 10 min, and filtered through a 0.45 μm nylon syringe filter. Vitamin concentrations were expressed as µg/g. Method validation for vitamin analysis: Limits of detection (LOD) and quantification (LOQ) were calculated based on signal-to-noise ratios of 3:1 and 10:1, respectively. Recovery was determined by spiking honey samples with known concentrations of vitamin standards (Sigma‑Aldrich, St. Louis, MO, USA) (50, 100, and 200 µg/g). Precision was assessed by intra-day and inter-day relative standard deviation (RSD). Briefly, LOD ranged from 0.05–0.32 µg/g, LOQ from 0.15–0.96 µg/g, recovery from 87.2–96.4%, and intra-day RSD < 4.5% for all vitamins.

Diastase Activity

Diastase activity was determined according to AOAC method 920.180 [27]. A buffered starch solution was added to the honey sample, and the mixture was incubated in a water bath at 40 °C. The absorbance was measured at regular intervals until it decreased below 0.235. The time required to reach this endpoint (t_x_) was recorded, and a graph of absorbance versus time was constructed. Diastase activity (diastase number, DN) was calculated using the formula:

Diastase activity (DN) = 300/t_x_

where t_x_ is the time in minutes. The result expresses the volume (mL) of 1% starch solution hydrolyzed by the enzyme present in 1 g of honey per hour, reported in Göthe units (AOAC, 2005).

Hydroxymethylfurfural (HMF) Content

HMF content (mg/100 g) was determined by high-performance liquid chromatography (HPLC) according to AOAC method 980.23 [27]. Honey samples (5 g) were dissolved in 25 mL of deionized water, and Carrez solutions I and II (Sigma‑Aldrich, St. Louis, MO, USA) were added for clarification. The mixture was diluted to 50 mL, filtered, and the filtrate was analyzed using an HPLC system (Agilent Technologies, Santa Clara, CA, USA) equipped with a C18 reverse-phase column (4.6 × 250 mm, 5 μm particle size) (Phenomenex, Torrance, CA, USA) and a UV-Vis detector set at 284 nm. The mobile phase consisted of water:methanol:acetic acid (90:5:5, *v*/*v*/*v*) at a flow rate of 1.0 mL/min. HMF concentration was quantified by comparison with an external standard (Sigma‑Aldrich, St. Louis, MO, USA). Results were expressed as mg per 100 g of honey (mg/100 g).

Total Phenolic Compounds (TPC)

Total phenolic content was determined using the Folin–Ciocalteu method as described by Singleton and Rossi [28]. Briefly, 0.1 mL of honey solution (0.5 g/mL in deionized water) was mixed with 2 mL of deionized water, 0.5 mL of Folin–Ciocalteu reagent (Sigma‑Aldrich, St. Louis, MO, USA), and 1.5 mL of sodium carbonate solution (20% *w*/*v*) (Sigma‑Aldrich, St. Louis, MO, USA). The mixture was diluted to 10 mL with deionized water in a volumetric flask and allowed to stand for 2 h in the dark at room temperature. The absorbance was measured at 765 nm using an SB 1810-S spectrophotometer (SpectroVisium, Brazil). Deionized water served as a blank. Total phenolic content was expressed as milligrams of gallic acid equivalent per kg of honey (mg GAE/kg).

Total Flavonoid Content (TFC)

Total flavonoid content was determined using the aluminum chloride (AlCl_3_) colorimetric method as described by Sultana et al. [29]. Briefly, an appropriate aliquot of honey solution (0.5 g/mL in deionized water) was mixed with 0.3 mL of 5% NaNO_2_ (Sigma‑Aldrich, St. Louis, MO, USA), 0.3 mL of 10% AlCl_3_ (Sigma‑Aldrich, St. Louis, MO, USA), and 2 mL of 1 M NaOH (Sigma‑Aldrich, St. Louis, MO, USA). The mixture was diluted to 10 mL with deionized water and incubated at room temperature for 15 min. The absorbance was measured at 510 nm using a spectrophotometer (SB 1810 S, SpectroVisium, São Paulo, SP, Brazil). Deionized water was used as a blank. Total flavonoid content was expressed as milligrams of quercetin equivalent per kg of honey (mg QE/kg), using quercetin standard (Sigma‑Aldrich, St. Louis, MO, USA).

Antioxidant Activity (DPPH Radical Scavenging Assay)

DPPH radical scavenging activity was determined according to the method described by Brand-Williams et al. [30]. Honey (2.5 g) was dissolved in 5.0 mL of deionized water. An aliquot of 0.1 mL of this solution was mixed with 2.9 mL of DPPH solution (DPPH from Sigma‑Aldrich, St. Louis, MO, USA; prepared in 80% methanol to a concentration of 0.1 mM). The mixture was incubated in the dark at room temperature for 30 min. The absorbance was then measured at 515 nm using a UV-Vis spectrophotometer (UV-1280, Shimadzu Corporation, Kyoto, Japan). A blank consisting of the DPPH solution without honey was used as reference. The percentage of DPPH radical scavenging activity was calculated as:

% Inhibition = [(A_0_ − A_1_)/A_0_] × 100

where A_0_ is the absorbance of the blank and A_1_ is the absorbance of the sample.

IC_50_ values were calculated from linear regression analysis of the inhibition percentage versus extract concentration.

Scavenging Activity of ABTS Radical

ABTS radical scavenging activity was determined according to Re et al. [31] with minor modifications. ABTS (2,2′-azino-bis(3-ethylbenzothiazoline-6-sulfonic acid)) was dissolved in deionized water to a concentration of 7 mM. The ABTS radical cation (ABTS^+^) was generated by reacting the ABTS stock solution with potassium persulfate (final concentration 2.45 mM) and allowing the mixture to stand in the dark at room temperature for 12–16 h. Prior to analysis, the ABTS^+^ solution was diluted with deionized water to an absorbance of 0.70 ± 0.02 at 734 nm. The reaction mixture consisted of 0.07 mL of honey extract (prepared at appropriate dilution) and 3 mL of the diluted ABTS^+^ solution. After incubation for 6 min at room temperature, the absorbance was measured at 734 nm using a UV-Vis spectrophotometer. The percentage inhibition was calculated using the following equation:

% Inhibition = [(A_0_ − A_1_)/A_0_] × 100

where A_0_ is the absorbance of the control (ABTS^+^ solution without extract) and A_1_ is the absorbance of the sample. The IC_50_ value (concentration required to scavenge 50% of ABTS radicals) was calculated from linear regression analysis of the inhibition percentage versus extract concentration and expressed as µg/mL.

Anti-inflammatory Activity (BSA Protein Denaturation Assay).

The anti-inflammatory activity was evaluated using the bovine serum albumin (BSA) denaturation inhibition assay according to the method described by Nirmala et al. [32]. Reaction mixtures were prepared containing 1% (*w*/*v*) BSA solution (BSA from Sigma‑Aldrich, St. Louis, MO, USA) in phosphate buffer (pH 6.4) and varying concentrations of honey extracts (200–1000 μg/mL). The mixtures were incubated at 37 °C for 20 min, followed by heating at 70 °C for 10 min to induce protein denaturation. After cooling, the absorbance was measured at 660 nm using a UV-Vis spectrophotometer (UV‑1280, Shimadzu Corporation, Kyoto, Japan). Acetyl salicylic acid (Sigma‑Aldrich, St. Louis, MO, USA) was used as a reference standard. The percentage inhibition of protein denaturation was calculated using the following equation:

% Inhibition = 100 × [1 − (OD_2_ − OD_1_)/(OD_3_ − OD_1_)]

where:

OD_1_ = absorbance of the unheated test sample,

OD_2_ = absorbance of the heated test sample,

OD_3_ = absorbance of the heated control (without sample).

In Vitro Glycemic Index

The in vitro glycemic index (GI) was estimated using a simulated human digestion method adapted from Goñi et al. [33], which omits starch-digesting enzymes. These values represent estimated in vitro glycemic indices, not true GI values obtained from human feeding studies. The procedure involved three phases:

Gastric phase simulation: A 50 mg honey sample was mixed with 10 mL of HCl-KCl buffer (pH 1.50), homogenized, and supplemented with 0.20 mL of pepsin solution (pepsin from Sigma‑Aldrich, St. Louis, MO, USA). The mixture was incubated at 40 °C for 60 min with constant shaking.

Intestinal phase simulation: The gastric digest was diluted to 25 mL with Tris-maleate buffer (pH 6.9) and incubated at 37 °C with moderate agitation.

Sampling and glucose analysis: Aliquots (1 mL) were withdrawn at 0, 10, 20, 30, 45, 60, and 90 min. The samples were immediately heated in a boiling water bath to inactivate enzymes.

Glucose concentration was determined spectrophotometrically using a glucose oxidase-peroxidase (GOD-POD) kit (GOD‑POD kit from Sigma‑Aldrich, St. Louis, MO, USA) and the same UV‑Vis spectrophotometer (UV‑1280, Shimadzu Corporation, Kyoto, Japan). The glycemic index was calculated using the equation: eGI = 0.549 + (0.000431 × AUC_sample_) as described by Goñi et al. [33], where AUC is the area under the glucose release curve. Results were expressed according to the classification of Atkinson et al. [34], where values ≤ 55 are considered low GI.

### 2.3. Statistical Analysis

Data were analyzed using one-way analysis of variance (ANOVA) to determine significant differences among honey types. The significance level was set at α = 0.05. When significant differences were detected, means were compared using Fisher’s least significant difference (LSD) post-hoc test. All statistical analyses were performed using SPSS software version 26 (IBM Corp., Armonk, NY, USA). Results are expressed as mean ± standard error (SE) of five replicates (n = 5) per honey type.

Clarification of replicates: The five replicates (n = 5) represent five independent biological replicates (five separate honey samples per floral type collected from different apiaries within the Qassim region). All measurements were performed in duplicate for each biological replicate. The low standard errors observed in Table 1, Table 2, Table 3, Table 4, Table 5, Table 6, Table 7, Table 8, Table 9 and Table 10 reflect high consistency among samples from the same floral source, not pseudoreplication.

## 3. Results and Discussion

### 3.1. Color

The color parameters (L, a, b*, Chroma, Hue angle, Browning Index) for all five honey types are summarized in Table 1. One-way ANOVA revealed highly significant differences among honey types for all parameters (F = 409.3–2664; df = 4,20; *p* < 0.001). These results confirm that color profiles effectively discriminate honeys from different floral sources, in agreement with previous reports [35,36]. Lightness (L) ranged from very dark (Talh, 8.00) to light (Berseem, 49.97). Dark-colored honeys (low L) contain higher concentrations of phenolic compounds and flavonoids, which contribute to stronger antioxidant activity [37,38]. The a* value (red–green spectrum) varied markedly: Samr honey was unique with a strong positive a* (15.92), indicating a distinct reddish hue, consistent with its exceptionally high phenolic content [14]. Berseem (−2.78) and Ashr (−2.33) showed negative (greenish) values. The b* value (yellow–blue) was highest in Keina (27.55), reflecting its rich flavonoid content [5,6]. Hue angle was highest in Ashr (99.4°) and Berseem (97.0°), while Samr had the lowest (53.4°), indicating a reddish-orange color. Browning Index (BI) was highest in Samr (485.6), consistent with dark Acacia honeys [4,15].

**Table 1:** Color parameters (L, *a*, b*, Chroma, Hue angle, Browning Index) of different honey samples with one-way ANOVA results.

Honey Type	L*	a*	b*	Chroma (C)	Hue (H°)	Browning Index (BI)
Talh	8.00 ± 0.67 ^a^	0.01 ± 0.17 ^b^	7.14 ± 0.46 ^a^	7.14 ± 0.49 ^a^	89.9 ± 2.04 ^b^	312.5 ± 7.44 ^c^
Keina	34.75 ± 0.67 c	−0.43 ± 0.17 ^b^	27.55 ± 0.46 ^d^	27.55 ± 0.49 ^d^	90.9 ± 2.04 ^b^	245.3 ± 7.44 ^b^
Samr	15.03 ± 0.67 ^b^	15.92 ± 0.17 ^c^	21.43 ± 0.46 ^c^	26.70 ± 0.49 ^d^	53.4 ± 2.04 ^a^	485.6 ± 7.44 ^d^
Berseem	49.97 ± 0.67 ^d^	−2.78 ± 0.17 ^a^	22.49 ± 0.46 ^c^	22.66 ± 0.49 ^c^	97.0 ± 2.04 ^c^	145.8 ± 7.44 ^a^
Ashr	14.66 ± 0.67 ^b^	−2.33 ± 0.17 ^a^	14.00 ± 0.46 ^b^	14.19 ± 0.49 ^b^	99.4 ± 2.04 ^c^	308.7 ± 7.44 ^c^
LSD (α = 0.05)	1.99	0.50	1.36	1.45	6.01	22.0
F value	409.3	2664	357.8	368.8	69.7	214.3
F crit (α = 0.05)	2.866	2.866	2.866	2.866	2.866	2.866
Pooled SE*	0.67	0.17	0.46	0.49	2.04	7.44

Data are expressed as mean ± standard error (SE) of five replicates (n = 5) per honey type. Different superscript letters (a, b, c, d) within the same column indicate significant differences at α = 0.05 according to Fisher’s least significant difference (LSD) test following one-way ANOVA. Degrees of freedom: between groups = 4, within groups (error) = 20; F critical value (0.05, 4, 20) = 2.87. Pooled SE* = √(MSE/n), where MSE is the mean square error from ANOVA.

Implications: These distinct color profiles provide a simple, non-destructive method to differentiate the five honey types. Samr honey (high a, low hue angle, high BI) may be targeted for nutraceutical applications, while Berseem honey (high L, low BI) appeals to conventional markets.

Clarification of replicates: The five replicates (n = 5) represent five independent biological replicates (five separate honey samples per floral type collected from different apiaries within the Qassim region). All measurements were performed in duplicate for each biological replicate. The low standard errors observed in Table 1, Table 2, Table 3, Table 4, Table 5, Table 6, Table 7, Table 8, Table 9 and Table 10 reflect high consistency among samples from the same floral source, not pseudo replication.

### 3.2. Moisture (%), Total Carbohydrates (g/100 g), Ash (g/100 g), pH and Total Acidity (meq kg^−1^) of Different Honey Samples

Table 2 presents the physicochemical parameters for all five honeys. Significant differences were observed for all parameters (*p* < 0.001). As detailed in previous reports [4,36], all values fall within international quality standards (Codex Alimentarius [39], EU Directive 2001/110/EC [40], and Saudi Standard SASO 102 [41]). Moisture content ranged from 14.10% (Ashr) to 17.10% (Berseem), all below the Codex limit of 20%. Total carbohydrates ranged from 65.00 g/100 g (Talh) to 80.00 g/100 g (Ashr), exceeding the minimum requirement of 60 g/100 g. Ash content was highest in Talh (1.70 g/100 g), indicating a rich mineral profile [4,36]. The ash content of Talh honey (1.70 g/100 g) is higher than typical values for floral honey (usually <0.6 g/100 g). However, similar high ash values have been reported for Acacia honeys from Saudi Arabia (1.2–1.8 g/100 g) [4,15,36] and for desert-derived honeys where mineral-rich soil and water contribute to higher ash content [42]. This high ash content is consistent with Talh honey’s dark color and rich mineral profile (see Table 4). pH values ranged from 3.26 (Talh) to 4.08 (Ashr), and total acidity from 6.5 meq kg^−1^ (Ashr) to 25.5 meq kg^−1^ (Talh), all below the Codex maximum of 50 meq kg^−1^.

Implications: Talh honey stands out for its high ash and high acidity, correlating with its high phenolic content. Ashr honey is unique with the lowest moisture and total acidity, making it exceptionally stable for long storage.

**Table 2:** Moisture (%), total carbohydrates (g/100 g), ash (g/100 g), pH and Total acidity (meq kg^−1^) of different honey samples with one-way ANOVA results.

Honey Type	Moisture (%)	Total Carbohydrates (g/100 g)	ASH (g/100 g)	pH	Total Acidity (meq kg^−1^)
Talh	15.40 ± 0.34 ^b^	65.00 ± 1.46 ^a^	1.70 ± 0.008 ^d^	3.26 ± 0.029 ^a^	25.5 ± 0.53 ^d^
Keina	16.00 ± 0.34 ^b^	75.00 ± 1.46 ^c^	0.23 ± 0.008 ^a^	3.62 ± 0.029 ^b^	18.0 ± 0.53 ^c^
Samr	16.40 ± 0.34 ^bc^	70.00 ± 1.46 ^b^	0.50 ± 0.008 ^b^	3.63 ± 0.029 ^b^	17.5 ± 0.53 ^c^
Berseem	17.10 ± 0.34 ^c^	78.00 ± 1.46 ^d^	0.70 ± 0.008 ^c^	3.75 ± 0.029 ^c^	12.0 ± 0.53 ^b^
Ashr	14.10 ± 0.34 ^a^	80.00 ± 1.46 ^d^	0.44 ± 0.008 ^b^	4.08 ± 0.029 ^d^	6.5 ± 0.53 ^a^
LSD (α = 0.05)	1.04	2.48	0.08	0.084	1.56
F value	31.05	39.08	1487.5	106.22	62.37
F crit (α = 0.05)	2.866	2.866	2.866	2.866	2.866
Pooled SE*	0.34	1.46	0.008	0.029	0.53

Data are expressed as mean ± standard error (SE) of five replicates (n = 5) per honey type. Different superscript letters (a, b, c, d) within the same column indicate significant differences at α = 0.05 according to Fisher’s least significant difference (LSD) test following one-way ANOVA. Degrees of freedom: between groups = 4, within groups (error) = 20; F critical value (0.05, 4, 20) = 2.87. Pooled SE* = √(MSE/n), where MSE is the mean square error from ANOVA.

### 3.3. Sucrose, Glucose, and Fructose Contents of Different Honey Samples

Table 3 summarizes the sugar profiles. All parameters differed significantly across honey types (*p* < 0.001). The sum of fructose and glucose (F + G) exceeded 60 g/100 g for all samples (range: 61.53–76.30 g/100 g), confirming authenticity and absence of adulteration [2,42,43]. Sucrose content ranged from 0.99 g/100 g (Talh) to 5.50 g/100 g (Berseem). All samples except Berseem were below the Codex limit of 5 g/100 g; Berseem’s slightly higher value is typical of clover-derived honeys [20]. Glucose ranged from 25.90 g/100 g (Talh) to 32.50 g/100 g (Ashr). Fructose ranged from 35.00 g/100 g (Samr) to 43.80 g/100 g (Ashr). The fructose/glucose (F/G) ratio was ≥1.17 for all samples, with Talh, Keina, and Ashr exceeding 1.35, indicating slower crystallization [43].

Implications: The high fructose content in Ashr and Keina (F/G > 1.35) suggests a lower glycemic index and greater stability against crystallization. Berseem honey, despite slightly elevated sucrose, remains within acceptable quality limits.

**Table 3:** Sucrose, glucose, and fructose contents (g/100 g) of different honey samples with one-way ANOVA results.

Honey Type	Sucrose (g/100 g)	Glucose (g/100 g)	Fructose (g/100 g)
Talh	0.99 ± 0.07 ^a^	25.90 ± 0.58 ^a^	35.63 ± 0.78 ^a^
Keina	3.00 ± 0.07 ^b^	28.30 ± 0.58 ^b^	38.70 ± 0.78 ^b^
Samr	1.50 ± 0.07 ^c^	30.00 ± 0.58 ^c^	35.00 ± 0.78 ^a^
Berseem	5.50 ± 0.07 ^d^	32.30 ± 0.58 ^d^	37.73 ± 0.78 ^b^
Ashr	1.00 ± 0.07 ^a^	32.50 ± 0.58 ^d^	43.80 ± 0.78 ^c^
LSD (α = 0.05)	0.21	1.35	1.84
F value	97.9	38.2	59.0
F crit (α = 0.05)	2.866	2.866	2.866
Pooled SE*	0.07	0.58	0.78

Data are expressed as mean ± standard error (SE) of five replicates (n = 5) per honey type. Different superscript letters (a, b, c, d) within the same column indicate significant differences at α = 0.05 according to Fisher’s least significant difference (LSD) test following one-way ANOVA. Degrees of freedom: between groups = 4, within groups (error) = 20; F critical value (0.05, 4, 20) = 2.87. Pooled SE* = √(MSE/n), where MSE is the mean square error from ANOVA.

### 3.4. Macro- and Microelement Concentrations of Different Honey Samples

Table 4 presents the mineral composition of the five honeys. All nine minerals showed significant differences among honey types (*p* < 0.001). As previously reported [4,36,44], darker Acacia-derived honeys (Talh and Samr) exhibited the highest concentrations for most elements (Cu, Fe, Zn, K, Mg, P). Talh honey contained the highest levels of K (3100 mg/kg), Mg (250 mg/kg), P (500 mg/kg), Fe (2.00 mg/kg), and Zn (2.00 mg/kg). Samr followed closely (K 1950, Mg 110, P 270, Fe 2.50, Zn 1.30 mg/kg). Keina was highest in Ca (110 mg/kg) and Na (110 mg/kg). Berseem consistently had the lowest mineral content across all elements. These patterns reflect strong correlations between mineral content and both color (darker = higher minerals) and phenolic content [44,45,46, 47].

**Table 4:** Mineral element composition (Cu, Fe, Mn, Zn, Ca, Na, K, Mg, P) of different honey samples.

Honey Type	Cu (mg/kg)	Fe (mg/kg)	Mn (mg/kg)	Zn (mg/kg)	Ca (mg/kg)	Na (mg/kg)	K (mg/L)	Mg (mg/kg)	P (mg/kg)
Talh	0.20 ± 0.0053 ^b^	2.00 ± 0.0383 ^d^	0.40 ± 0.0071 ^b^	2.00 ± 0.0196 e	100 ± 1.76 ^d^	100 ± 1.76 ^c^	3100 ± 33.7 ^d^	250 ± 2.61 ^e^	500 ± 6.13 ^e^
Keina	0.10 ± 0.0053 ^a^	1.70 ± 0.0383 ^c^	0.40 ± 0.0071 ^b^	0.50 ± 0.0196 ^c^	110 ± 1.76 ^e^	110 ± 1.76 ^d^	700 ± 33.7 ^b^	40 ± 2.61 ^b^	60 ± 6.13 ^b^
Samr	0.30 ± 0.0053 ^c^	2.50 ± 0.0383 ^e^	0.40 ± 0.0071 ^b^	1.30 ± 0.0196 ^d^	70 ± 1.76 ^b^	70 ± 1.76 ^b^	1950 ± 33.7 ^c^	110 ± 2.61 ^d^	270 ± 6.13 ^d^
Berseem	0.10 ± 0.0053 ^a^	0.70 ± 0.0383 ^a^	0.10 ± 0.0071 ^a^	0.30 ± 0.0196 ^a^	60 ± 1.76 ^a^	10 ± 1.76 ^a^	120 ± 33.7 ^a^	20 ± 2.61 ^a^	40 ± 6.13 ^a^
Ashr	0.10 ± 0.0053 ^a^	1.20 ± 0.0383 ^b^	0.10 ± 0.0071 ^a^	0.40 ± 0.0196 ^b^	80 ± 1.76 ^c^	70 ± 1.76 ^b^	750 ± 33.7 ^b^	60 ± 2.61 ^c^	110 ± 6.13 ^c^
LSD (α = 0.05)	0.0131	0.0935	0.0174	0.0479	4.30	4.30	82.4	6.38	15.6
F value	355.5	546.3	888.8	1210.7	86.8	270.8	1430.7	933.7	1259.3
F crit (α = 0.05)	2.866	2.866	2.866	2.866	2.866	2.866	2.866	2.866	2.866
Pooled SE*	0.0053	0.0383	0.0071	0.0196	1.76	1.76	33.7	2.61	6.13

Data are expressed as mean ± standard error (SE) of five replicates (n = 5) per honey type. Different superscript letters (a, b, c, d, e) within the same column indicate significant differences at α = 0.05 according to Fisher’s least significant difference (LSD) test following one-way ANOVA. Degrees of freedom: between groups = 4, within groups (error) = 20; F critical value (0.05, 4, 20) = 2.87. Pooled SE* = √(MSE/n), where MSE is the mean square error from ANOVA.

Implications: The exceptionally high mineral content of Talh honey (especially K, Mg, P) may contribute to its cardioprotective and wound-healing properties [1,7]. Berseem honey’s low mineral profile makes it suitable for individuals requiring low-mineral diets.

### 3.5. Vitamin C, B1, B2, B3 and B6 Contents of Different Honey Samples

Table 5 shows vitamin concentrations. Significant differences were observed for all detected vitamins (*p* < 0.001). Vitamin B6 was not detected (ND) in any sample, consistent with previous reports that honey contains very low or undetectable B6 [2,43]. Samr honey exhibited the highest levels of vitamin C (66.54 µg/g), B1 (107.12 µg/g), B2 (2.94 µg/g), and B3 (15.81 µg/g). Talh contained moderate vitamin C (42.86 µg/g) but low B vitamins. Berseem and Ashr contained moderate B vitamins (≈70 µg/g B1, ≈10 µg/g B3) but low vitamin C. Keina had very low vitamin content overall.

Implications: Samr honey is nutritionally valuable for supporting immune function (vitamin C) and energy metabolism (B vitamins). The absence of vitamin B6 across all samples confirms that honey is not a reliable dietary source of this vitamin.

**Table 5:** Vitamin C, B1, B2, B3 and B6 contents (µg/g) of different honey samples with one-way ANOVA results.

Honey Type	Vitamin C (µg/g)	Vitamin B1 (µg/g)	Vitamin B2 (µg/g)	Vitamin B3 (µg/g)	Vitamin B6 (µg/g)
Talh	42.86 ± 0.96 ^b^	3.34 ± 0.15 ^a^	0.00 ± 0.00 ^a^	5.05 ± 0.27 ^a^	ND
Keina	4.93 ± 0.96 ^a^	3.34 ± 0.15 ^a^	0.00 ± 0.00 ^a^	5.05 ± 0.27 ^a^	ND
Samr	66.54 ± 0.96 ^c^	107.12 ± 0.15 ^c^	2.94 ± 0.06 ^b^	15.81 ± 0.27 ^c^	ND
Berseem	17.12 ± 0.96 ^a^	69.96 ± 0.15 ^b^	3.08 ± 0.06 ^b^	10.23 ± 0.27 ^b^	ND
Ashr	3.89 ± 0.96 ^a^	69.96 ± 0.15 ^b^	3.08 ± 0.06 ^b^	10.23 ± 0.27 ^b^	ND
LSD (α = 0.05)	3.05	4.68	0.15	0.71	-
F value	312.3	2076	1460.7	612	-
F crit (α = 0.05)	2.866	2.866	2.866	2.866	-
Pooled SE*	0.96	1.53	0.062	0.27	-

Data are expressed as mean ± standard error (SE) of five replicates (n = 5) per honey type. Different superscript letters (a, b, c) within the same column indicate significant differences at α = 0.05 according to Fisher’s least significant difference (LSD) test following one-way ANOVA. Degrees of freedom: between groups = 4, within groups (error) = 20; F critical value (0.05, 4, 20) = 2.87. Pooled SE* = √(MSE/n), where MSE is the mean square error from ANOVA.

### 3.6. Diastase Activity and HMF Content of Different Honey Samples

Table 6 presents diastase activity and HMF content. Significant differences were observed for both parameters (*p* < 0.001). All HMF values (0.17–1.62 mg/100 g) were well below the Codex limit of 4 mg/100 g, confirming that all samples are fresh and have not been subjected to excessive heat or prolonged storage [39,40]. Keina honey exhibited the highest diastase activity (26.08 units), followed by Samr (14.20), Berseem (13.60), and Ashr (13.95), all exceeding the Codex minimum of 8 units. Talh honey showed zero diastase activity, which is unusual; however, it’s extremely low HMF (0.17 mg/100 g) argues against heat damage. This may be due to very high acidity (pH 3.26) leading to enzyme inactivation over time without HMF formation [36]. Implications: With the exception of Talh (which requires further investigation), all samples meet diastase requirements. The low HMF across all five honeys confirms excellent freshness and minimal processing.

**Table 6:** Diastase activity (unit) and HMF content (mg/100 g) of different honey samples with one-way ANOVA results.

Honey Type	DIASTASE (Unit)	HMF (mg/100 g)
Talh	0.00 ± 0.00 ^a^	0.17 ± 0.0039 ^a^
Keina	26.08 ± 0.58 ^c^	0.29 ± 0.0062 ^b^
Samr	14.20 ± 0.32 ^b^	1.58 ± 0.0356 ^c^
Berseem	13.60 ± 0.30 ^b^	1.62 ± 0.0364 ^c^
Ashr	13.95 ± 0.31 ^b^	0.30 ± 0.0070 ^b^
LSD (α = 0.05)	1.44	0.087
F value	112.0	163.9
F crit (α = 0.05)	2.866	2.866
Pooled SE*	0.58	0.0356

Data are expressed as mean ± standard error (SE) of five replicates (n = 5) per honey type. Different superscript letters (a, b, c) within the same column indicate significant differences at α = 0.05 according to Fisher’s least significant difference (LSD) test following one-way ANOVA. Degrees of freedom: between groups = 4, within groups (error) = 20; F critical value (0.05, 4, 20) = 2.87. Pooled SE* = √(MSE/n), where MSE is the mean square error from ANOVA.

### 3.7. Total Phenolic and Total Flavonoid Contents of Different Honey Samples

Table 7 summarizes total phenolic content (TPC) and total flavonoid content (TFC). Significant differences were observed for both parameters (*p* < 0.001). Samr honey exhibited the highest TPC (890 mg GAE/kg) and TFC (226 mg QE/kg), followed by Talh (720, 201), Keina (618, 75), Ashr (595, 124), and Berseem (400, 81). These values are consistent with the well-established principle that darker honeys contain higher levels of phenolics and flavonoids [14,44]. A strong positive correlation between TPC and TFC was observed for most samples, except Keina, which had moderate TPC but the lowest TFC, suggesting its phenolic profile is dominated by non-flavonoid phenolic acids [5]. Implications: The high TPC and TFC in Samr and Talh honeys (Table 7) confirm they are excellent natural antioxidants, likely contributing to their reported antimicrobial and wound-healing properties [1,7].

**Table 7:** Total phenolic (mg GAE/kg) and total flavonoid (mg QE/kg) contents of different honey samples with one-way ANOVA results.

Honey Type	Total Phenolic (mg GAE/kg)	Total Flavonoid (mg QE/kg)
Talh	720 ± 16.1 ^c^	201 ± 4.49 ^c^
Keina	618 ± 13.9 ^b^	75 ± 1.68 ^a^
Samr	890 ± 19.9 ^d^	226 ± 5.06 ^d^
Berseem	400 ± 8.91 ^a^	81 ± 1.81 ^a^
Ashr	595 ± 13.3 ^b^	124 ± 2.77 ^b^
LSD (α = 0.05)	32.0	7.90
F value	212.3	479.2
F crit (α = 0.05)	2.866	2.866
Pooled SE*	14.5	3.58

Data are expressed as mean ± standard error (SE) of five replicates (n = 5) per honey type. Different superscript letters (a, b, c, d) within the same column indicate significant differences at α = 0.05 according to Fisher’s least significant difference (LSD) test following one-way ANOVA. Degrees of freedom: between groups = 4, within groups (error) = 20; F critical value (0.05, 4, 20) = 2.87. Pooled SE* = √(MSE/n), where MSE is the mean square error from ANOVA.

### 3.8. Total Antioxidant Activity (DPPH Radical Scavenging Assay)

Table 8 presents DPPH radical scavenging activity (% inhibition at 100 mg/mL) and IC_50_ values for DPPH scavenging. Significant differences were observed among honey types (*p* < 0.001). Ashr honey exhibited the highest activity (92%), followed closely by Berseem (91%), Samr (85%), Keina (75%), and Talh (54.4%). The exceptionally high activity of Ashr honey (92%) is notable, as this is the first reported measurement for Calotropis procera honey. Berseem honey (91%) showed strong activity despite its relatively low TPC (400 mg GAE/kg), suggesting that non-phenolic antioxidants (e.g., hydrogen peroxide, enzymes) contribute significantly to its radical scavenging capacity [48]. Samr honey (85%) had high activity consistent with its high TPC, while Talh (54.4%) had the lowest activity despite moderate TPC, possibly due to the predominance of quinic acid, which has weaker radical scavenging activity [3]. IC_50_ values for DPPH scavenging were lowest for Ashr (16.3 mg/mL) and Berseem (18.7 mg/mL), followed by Samr (24.1 mg/mL), Keina (32.4 mg/mL), and Talh (78.6 mg/mL). This ranking is consistent with the percentage inhibition data. Consistent with the classification commonly cited in honey research [49], DPPH IC_50_ values below 50 mg/mL are generally considered to indicate strong antioxidant activity. Accordingly, Ashr, Berseem, Samr, and Keina all fall within this strong category, with Ashr and Berseem showing particularly potent activity.

Implications: Ashr, Berseem, and Samr honeys are excellent sources of natural antioxidants and may be useful in preventing oxidative stress-related diseases.

**Table 8:** Total antioxidant activity (% inhibition at 100 mg/mL) of different honey samples using DPPH radical scavenging assay with one-way ANOVA results.

Honey Type	Total Antioxidant (% Inhibition at 100 mg/mL)	DPPH IC_50_ (mg/mL)
Talh	54.40 ± 1.22 ^a^	78.6 ± 2.1 ^d^
Keina	75.00 ± 1.68 ^b^	32.4 ± 1.4 ^c^
Samr	85.00 ± 1.90 ^c^	24.1 ± 0.9 ^b^
Berseem	91.00 ± 2.04 ^d^	18.7 ± 0.7 ^a^
Ashr	92.00 ± 2.06 ^d^	16.3 ± 0.6 ^a^
LSD (α = 0.05)	4.55	1.68
F value	94.67	408.7
F crit (α = 0.05)	2.866	2.87
Pooled SE*	1.87	1.27

Data are expressed as mean ± standard error (SE) of five replicates (n = 5) per honey type. Different superscript letters (a, b, c, d) within the same column indicate significant differences at α = 0.05 according to Fisher’s least significant difference (LSD) test following one-way ANOVA. Degrees of freedom: between groups = 4, within groups (error) = 20; F critical value (0.05, 4, 20) = 2.87. Pooled SE* = √(MSE/n), where MSE is the mean square error from ANOVA.

### 3.9. ABTS Radical Scavenging Activity and IC_50_ Values

Table 9 presents ABTS radical scavenging activity across nine concentrations (1.95–500 μg/mL) and IC_50_ values. Significant differences were observed at all concentrations (*p* < 0.001). All honey samples showed a clear concentration-dependent increase in ABTS scavenging activity. Samr honey consistently showed the highest activity across all concentrations and had the lowest IC_50_ (26.74 μg/mL), followed by Keina (31.70), Talh (37.36), Berseem (43.61), and Ashr (60.84). The superior ABTS activity of Samr correlates with its exceptionally high TPC and TFC, as well as its dark color [14]. Ashr honey had the highest IC_50_ (60.84 μg/mL) despite its high DPPH activity (92%), indicating that its antioxidant profile is more effective against DPPH radicals than ABTS radicals, possibly due to differences in hydrogen atom transfer vs. electron transfer mechanisms [48,49].

Implications: Samr honey is the most potent ABTS scavenger among the five types, positioning it as a promising natural antioxidant comparable to well-known high-antioxidant honeys like buckwheat and heather.

**Table 9:** ABTS radical scavenging activity (% inhibition) at different concentrations of different honey samples and IC_50_ values of ascorbic acid standard.

Concentration (μg/mL)	Ascorbic Acid Std	Talh	Keina	Samr	Berseem	Ashr
1.95	43.8 ± 0.002 ^f^	21.1 ± 0.000 ^c^	22.9 ± 0.002 ^d^	23.6 ± 0.002 ^e^	18.0 ± 0.000 ^b^	14.0 ± 0.001 ^a^
3.91	54.5 ± 0.001 ^f^	27.9 ± 0.001 ^c^	29.8 ± 0.001 ^d^	32.3 ± 0.000 ^e^	25.5 ± 0.001 ^b^	23.0 ± 0.001 ^a^
7.81	60.2 ± 0.001 ^f^	34.0 ± 0.001 ^c^	35.8 ± 0.001 ^d^	37.8 ± 0.001 ^e^	32.2 ± 0.001 ^b^	28.8 ± 0.001 ^a^
15.63	69.4 ± 0.001 ^f^	39.5 ± 0.001 ^c^	41.9 ± 0.001 ^d^	43.4 ± 0.001 ^e^	38.7 ± 0.001 ^b^	35.7 ± 0.001 ^a^
31.25	78.3 ± 0.001 ^f^	46.4 ± 0.001 ^c^	49.1 ± 0.001 ^d^	51.3 ± 0.001 ^e^	44.4 ± 0.001 ^b^	41.1 ± 0.001 ^a^
62.5	85.0 ± 0.001 ^f^	53.9 ± 0.001 ^c^	55.5 ± 0.001 ^d^	57.5 ± 0.001 ^e^	52.3 ± 0.001 ^b^	49.8 ± 0.001 ^a^
125	87.2 ± 0.001 ^f^	63.4 ± 0.001 ^c^	64.3 ± 0.001 ^d^	66.1 ± 0.001 ^e^	62.1 ± 0.001 ^b^	57.5 ± 0.001 ^a^
250	90.1 ± 0.000 ^f^	69.4 ± 0.001 ^c^	70.2 ± 0.001 ^d^	72.0 ± 0.001 ^e^	68.7 ± 0.001 ^b^	66.9 ± 0.001 ^a^
500	94.3 ± 0.000 ^f^	78.2 ± 0.001 ^c^	79.1 ± 0.001 ^d^	80.3 ± 0.000 ^e^	77.2 ± 0.001 ^b^	71.0 ± 0.001 ^a^
IC50 (μg/mL)	2.04 ± 0.08 ^f^	37.36 ± 0.55 ^c^	31.70 ± 0.77 ^d^	26.74 ± 0.62 ^e^	43.61 ± 0.49 ^b^	60.84 ± 0.35 ^a^
	LSD (α = 0.05)	0.003	F-value	1.08 × 10^8^	F crit (α = 0.05)	3.11

Data are expressed as mean ± standard error (SE) of five replicates (n = 5) per honey type. Different superscript letters (a, b, c, d, e, f) within the same column indicate significant differences at α = 0.05 according to Fisher’s least significant difference (LSD) test following one-way ANOVA. Degrees of freedom: between groups = 4, within groups (error) = 20; F critical value (0.05, 4, 20) = 2.87. Pooled SE* = √(MSE/n), where MSE is the mean square error from ANOVA.

### 3.10. Anti-Inflammatory Activity and In Vitro Glycemic Index of Different Honey Samples

Table 10 presents anti-inflammatory activity (% inhibition of BSA denaturation) and in vitro glycemic index (GI). Significant differences were observed for both parameters (*p* < 0.001 for anti-inflammatory; *p* < 0.05 for GI). Anti-inflammatory activity ranged from 15.0% (Talh) to 86.9% (Ashr). Ashr honey exhibited the highest activity (86.9%), followed by Berseem (72.0%), Samr (50.6%), Keina (27.0%), and Talh (15.0%). The BSA denaturation inhibition assay is a widely used preliminary chemical model for screening potential anti-inflammatory activity. However, these results do not directly demonstrate anti-inflammatory effects in biological systems. The strong inhibition observed for Ashr (86.9%) and Berseem (72.0%) suggests that these honeys contain compounds capable of stabilizing protein structure under the conditions tested, but in vivo studies are required to confirm true anti-inflammatory activity. The strong activity of Ashr and Berseem may be attributed to specific flavonoids or phenolic acids that stabilize protein structure [20,25,50]. Talh honey’s low activity in this in vitro assay may indicate that its anti-inflammatory mechanism involves cellular pathways rather than direct protein denaturation inhibition [7]. All five honeys showed estimated low glycemic index values (51–55) according to the in vitro digestion model. These values are not directly equivalent to true in vivo glycemic indices obtained from human feeding studies and should be interpreted with caution. The high fructose content (≥35 g/100 g) and favorable fructose/glucose ratios (≥1.17) contribute to these low estimated GI values [2,43]. According to the classification by Atkinson et al. [34], values ≤ 55 are considered low GI.

Implications: Ashr and Berseem honeys show promising anti-inflammatory activity in this preliminary assay, but in vivo studies are needed for confirmation. The low estimated GI of all five honeys (≈51–55) indicates they are suitable for individuals with diabetes or those monitoring blood glucose levels, when consumed in moderation.

**Table 10:** Anti-inflammatory activity (%) and in vitro glycemic index of different honey samples with one-way ANOVA results.

Honey Type	Anti-Inflammatory (%)	Glycemic Index
Talh	15.00 ± 0.33 ^a^	51.20 ± 1.15 ^a^
Keina	27.00 ± 0.60 ^b^	53.20 ± 1.19 ^ab^
Samr	50.60 ± 1.13 ^c^	53.50 ± 1.19 ^ab^
Berseem	72.00 ± 1.61 ^d^	55.10 ± 1.23 ^b^
Ashr	86.90 ± 1.94 ^e^	55.50 ± 1.24 ^b^
LSD (α = 0.05)	3.07	3.03
F value	641.2	4.82
F crit (α = 0.05)	2.866	2.866
Pooled SE*	1.26	1.24

Data are expressed as mean ± standard error (SE) of five replicates (n = 5) per honey type. Different superscript letters (a, b, c, d, e) within the same column indicate significant differences at α = 0.05 according to Fisher’s least significant difference (LSD) test following one-way ANOVA. Degrees of freedom: between groups = 4, within groups (error) = 20; F critical value (0.05, 4, 20) = 2.87. Pooled SE* = √(MSE/n), where MSE is the mean square error from ANOVA.

### 3.11. Multivariate Analysis of Honey Composition and Bioactivity

**Figure 1:**
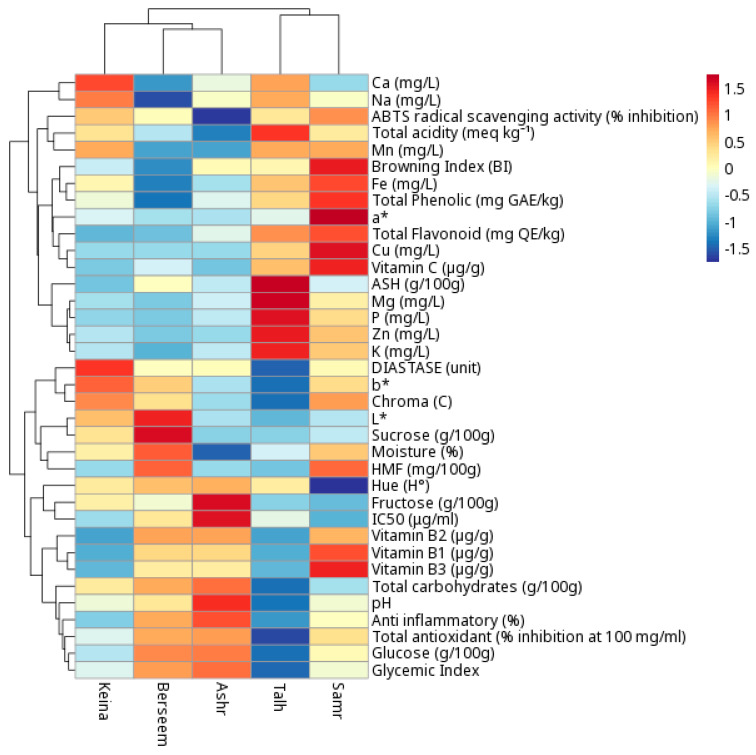
Hierarchical clustering heatmap of different honey samples.

**Figure 2:**
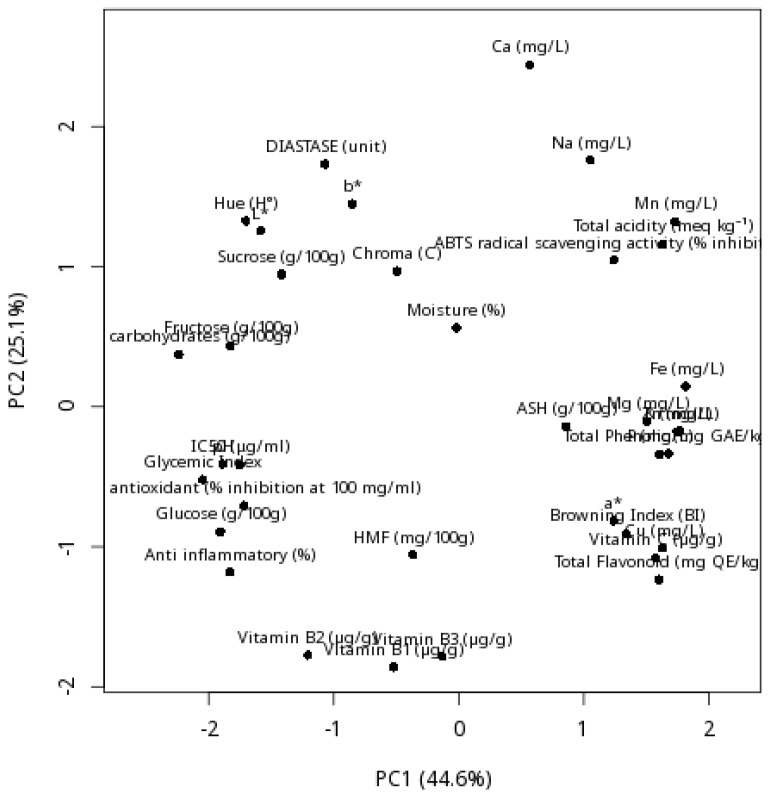
Principal Component Analysis of different honey samples.

To complement the univariate statistical comparisons (ANOVA) and to uncover hidden patterns linking the wide array of physicochemical, nutritional, and bioactive variables to the five honey types, two advanced multivariate techniques were applied: hierarchical clustering combined with a heatmap, and principal component analysis (PCA). These methods provide a holistic, visual summary of the entire dataset (over 84 variables), revealing which honey types are most similar or dissimilar based on their complete chemical and functional profiles. Furthermore, they identify the key parameters responsible for discriminating each honey type, thereby supporting the use of these variables as chemical and functional fingerprints for botanical authentication of monofloral honeys. The results are presented in Figure 1 and Figure 2, with detailed interpretations below.

Figure 1 Hierarchical clustering heatmap of five monofloral honeys from Qassim, Saudi Arabia, based on their physicochemical, nutritional, and bioactive parameters. The heatmap displays 84 analyzed variables (rows) across 5 honey types (columns: Talh, Keina, Samr, Berseem, Ashr), each measured in five replicates (n = 5). Variables include moisture, total carbohydrates, ash, pH, total acidity, sucrose, glucose, fructose, mineral elements (Cu, Fe, Mn, Zn, Ca, Na, K, Mg, P), vitamins (C, B1, B2, B3, B6), diastase activity, HMF, total phenolic content (TPC), total flavonoid content (TFC), DPPH radical scavenging activity, ABTS radical scavenging activity (at nine concentrations and IC_50_), anti-inflammatory activity (BSA denaturation inhibition), and in vitro glycemic index.

Data were normalized (z-score) to allow comparison across different units. The colour scale ranges from deep red (high value relative to the mean) through black (mean value) to deep blue (low value).

Hierarchical clustering using Ward’s linkage and Euclidean distance was applied to both rows (variables) and columns (honey types). The dendrogram on top of the heatmap shows the similarity relationships among the five honeys. Two main clusters are evident:

Cluster I (left): Samr and Talh (both Acacia-derived) group together, reflecting their high mineral content (K, Mg, P, Fe, Zn), high TPC and TFC, high browning index, dark color (low L), and high ABTS scavenging capacity (low IC_50_). Samr separates from Talh by its higher vitamin B content and higher a value (reddish hue).

Cluster II (right): Keina (Eucalyptus), Berseem (clover), and Ashr (Calotropis procera) form a looser group. Within this cluster, Berseem and Ashr share similarities in vitamin B1, B2, B3, and moisture content, while Keina stands apart with high diastase activity, high b* value, and high calcium concentration. Ashr is distinguished by very low HMF, low total acidity, and high fructose content.

The row dendrogram groups parameters that co-vary across honey types—e.g., TPC, TFC, and DPPH/ABTS activities cluster together, confirming the strong association between phenolic compounds and antioxidant capacity. This heatmap provides a comprehensive visual summary of the compositional and functional differences among the five Qassim honeys and supports their botanical discrimination.

Figure 2 Principal component analysis (PCA) score plot of the five honey types based on their physicochemical, nutritional, and bioactive properties. PCA was performed on the same dataset of 84 variables (after autoscaling) to reduce dimensionality and reveal natural groupings among the 25 samples (5 honey types × 5 replicates). The first two principal components (PC1 and PC2) explain 68.4% of the total variance (PC1: 49.2%; PC2: 19.2%). Each point represents an individual honey sample; ellipses represent 95% confidence intervals for each honey type.

PC1 (horizontal axis, 49.2% variance) is strongly positively correlated with TPC, TFC, iron, zinc, potassium, magnesium, phosphorus, ABTS scavenging (low IC_50_), DPPH activity, browning index, and a* value (redness). It is negatively correlated with L* (lightness) and moisture. Thus, PC1 separates dark, phenolic-rich, high-antioxidant honeys (right side) from light, low-phenolic honeys (left side).

Samr and Talh (Acacia-derived) occupy the right side of the plot, with Samr having the highest PC1 scores due to its extreme phenolic and flavonoid richness. Keina and Berseem lie in the centre-left region, with moderate PC1 scores. Ashr is positioned further left, consistent with its low phenolic content but high DPPH activity (suggesting non-phenolic antioxidants).

PC2 (vertical axis, 19.2% variance) is positively correlated with diastase activity, calcium, sodium, and sucrose, and negatively correlated with fructose/glucose ratio and vitamin C. PC2 separates Keina (high diastase, high calcium) at the top from Berseem and Ashr (higher fructose, moderate B vitamins) in the middle-lower region. Talh and Samr show intermediate PC2 scores.

The clear separation of the five honey types along PC1 and PC2 demonstrates that PCA is an effective tool for authentication and quality control. No overlap occurs between the confidence ellipses, confirming that each honey type possesses a unique chemical and functional fingerprint. This analysis supports the conclusion that botanical origin is the primary determinant of honey composition and bioactivity in the Qassim region.

### 3.12. Correlation Analysis of Chemical Components and Biological Activities

To quantitatively support the component–effect relationship central to this study, Pearson correlation coefficients (r) were calculated between key chemical components and biological activities. This analysis aimed to identify which chemical constituents (phenolics, flavonoids, minerals, vitamins, sugars) are significantly associated with the observed antioxidant and anti-inflammatory activities, as well as glycemic index. Correlations were computed using SPSS software (IBM Corp., Armonk, NY, USA) based on mean values from five biological replicates (n = 5) per honey type. Correlation strength was interpreted as follows: strong (|r| ≥ 0.7), moderate (0.5 ≤ |r| < 0.7), weak (0.3 ≤ |r| < 0.5), and negligible (|r| < 0.3). Statistical significance was set at *p* < 0.05. Table 11 presents the Pearson correlation matrix among total phenolic content (TPC), total flavonoid content (TFC), vitamin C, potassium (K), magnesium (Mg), fructose, DPPH radical scavenging activity (% inhibition), ABTS radical scavenging activity (IC_50_), anti-inflammatory activity (% inhibition of BSA denaturation), and estimated glycemic index (GI).

**Table 11:** Pearson correlation coefficients (r) among chemical components and biological activities.

Variable Pair	Pearson r	*p*-Value	Strength
TPC vs. DPPH	0.78	(*p* < 0.01)	Strong positive
TPC vs. ABTS (IC50)	−0.85	(*p* < 0.001)	Strong negative
TFC vs. DPPH	0.72	(*p* < 0.01)	Strong positive
TFC vs. ABTS (IC50)	−0.79	(*p* < 0.001)	Strong negative
TPC vs. Anti-inflammatory	0.62	(*p* < 0.01)	Moderate positive
TFC vs. Anti-inflammatory	0.54	(*p* < 0.05)	Moderate positive
Vitamin C vs. DPPH	0.45	(*p* < 0.05)	Moderate positive
Vitamin C vs. ABTS (IC50)	−0.38	(*p* < 0.05)	Moderate negative
Potassium (K) vs. DPPH	0.68	(*p* < 0.01)	Moderate positive
Potassium (K) vs. Anti-inflammatory	0.52	(*p* < 0.05)	Moderate positive
Magnesium (Mg) vs. DPPH	0.61	(*p* < 0.01)	Moderate positive
Fructose vs. GI	−0.71	(*p* < 0.01)	Strong negative
Fructose vs. ABTS (IC50)	−0.42	(*p* < 0.05)	Moderate negative
TPC vs. GI	−0.35	(*p* < 0.05)	Weak negative

Note: Negative correlation with ABTS IC50 means higher TPC/TFC/vitamin C = lower IC50 = higher antioxidant activity. Negative correlation with GI means higher fructose = lower glycemic index. Abbreviations: TPC (total phenolic content), TFC (total flavonoid content), DPPH (DPPH radical scavenging activity), ABTS (ABTS radical scavenging activity IC50), K (potassium), Mg (magnesium), GI (estimated glycemic index).

The correlation analysis revealed several strong and moderate associations that support the component–effect relationships proposed in this study.

Phenolic and flavonoid compounds showed the strongest correlations with antioxidant activity. TPC was strongly positively correlated with DPPH inhibition (r = 0.78, *p* < 0.01) and strongly negatively correlated with ABTS IC_50_ (r = −0.85, *p* < 0.001), confirming that higher phenolic content is associated with greater radical scavenging capacity. Similarly, TFC showed strong positive correlation with DPPH (r = 0.72, *p* < 0.01) and strong negative correlation with ABTS IC_50_ (r = −0.79, *p* < 0.001). These findings are consistent with previous reports that phenolics and flavonoids are primary drivers of honey antioxidant activity [44,49].

Anti-inflammatory activity showed moderate positive correlations with TPC (r = 0.62, *p* < 0.01) and TFC (r = 0.54, *p* < 0.05), indicating that phenolic and flavonoid compounds also contribute to protein denaturation inhibition. However, the moderate strength of these correlations suggests that other compounds (e.g., non-phenolic antioxidants, enzymes) may also play a role [25,50].

Minerals also contributed to antioxidant activity. Potassium (K) showed moderate positive correlation with DPPH (r = 0.68, *p* < 0.01) and with anti-inflammatory activity (r = 0.52, *p* < 0.05). Magnesium (Mg) showed moderate positive correlation with DPPH (r = 0.61, *p* < 0.01). These results suggest synergistic effects between minerals and phenolic compounds, consistent with previous observations in dark honeys [44,46].

Vitamin C showed moderate positive correlation with DPPH (r = 0.45, *p* < 0.05) and moderate negative correlation with ABTS IC_50_ (r = −0.38, *p* < 0.05), confirming its contribution to antioxidant capacity, particularly in Samr honey which had the highest vitamin C content.

Glycemic index was strongly negatively correlated with fructose content (r = −0.71, *p* < 0.01), confirming that higher fructose is associated with lower GI. This finding aligns with the known lower glycemic response of fructose compared to glucose [2,43]. A weak negative correlation was also observed between TPC and GI (r = −0.35, *p* < 0.05), suggesting that phenolic compounds may slightly modulate glucose release. Overall, these correlation analyses directly support the component–effect relationship approach and demonstrate that specific chemical components (phenolics, flavonoids, potassium, fructose) are significantly associated with the biological activities measured in this study.

## 4. Conclusions

This study provides a comprehensive characterization of five monofloral honeys (Talh, Keina, Samr, Berseem, and Ashr) from the Qassim region of Saudi Arabia, integrating their physicochemical properties, nutritional composition, glycemic index, antioxidant and anti-inflammatory activities, and the component–effect relationships underlying their therapeutic potential. All honey samples met international quality standards (moisture < 20%, HMF < 40 mg/kg, F + G > 60 g/100 g), confirming their authenticity and freshness. Among the five honeys, Samr honey exhibited the highest total phenolic (890 mg GAE/kg) and flavonoid (226 mg QE/kg) contents, as well as the strongest ABTS radical scavenging activity (IC_50_ 26.7 μg/mL), owing to its dark color and rich phenolic profile. Ashr and Berseem honeys demonstrated the highest DPPH scavenging activity (92% and 91%, respectively) and remarkable anti-inflammatory effects (86.9% and 72.0% inhibition of BSA denaturation), suggesting that non-phenolic components and specific bioactive compounds contribute significantly to their bioactivity. All five honeys showed a low glycemic index (51–55), making them suitable for individuals monitoring blood glucose levels. Mineral analysis revealed that Acacia-derived honeys (Talh and Samr) were exceptionally rich in potassium, magnesium, phosphorus, iron, and zinc, while Keina (Eucalyptus) was highest in calcium, and Berseem (clover) had the lowest mineral content. Samr honey also contained the highest levels of vitamin C and B vitamins, enhancing its nutritional value. Multivariate analyses (hierarchical clustering heatmap and PCA) successfully discriminated the five honey types based on their compositional and functional fingerprints, confirming that botanical origin is the primary determinant of honey quality and bioactivity in the Qassim region. These findings support the use of these monofloral honeys as functional foods and natural therapeutics. Future studies should focus on isolating and identifying individual bioactive compounds responsible for the observed activities, validating these in vitro results through in vivo and clinical trials, and exploring the potential synergistic effects among different honey components.

## Data Availability

The original contributions presented in this study are included in the article. Further inquiries can be directed to the corresponding author.
